# Relationship and Alliance Formation Processes in Psychotherapy: A Dual-Perspective Qualitative Study

**DOI:** 10.3389/fpsyg.2022.915932

**Published:** 2022-07-07

**Authors:** Kristina Osland Lavik, Andrew Athan McAleavey, Eli Karoline Kvendseth, Christian Moltu

**Affiliations:** ^1^Department of Psychiatry, District General Hospital of Førde, Førde, Norway; ^2^Department of Psychiatry, Weill Cornell Medicine, New York, NY, United States; ^3^Department of Health and Caring Sciences, Western Norway University of Applied Sciences, Bergen, Norway

**Keywords:** psychotherapy, therapeutic relationship processes, working alliance, real relationship, qualitative, interpersonal process recall

## Abstract

**Objective:**

To explore how therapists and clients act dyadically to establish a therapeutic relationship during the first five sessions of psychotherapy. The study aimed to identify both relational facilitative and hindering processes occurring in routine care.

**Methods:**

Using the method ‘interpersonal process recall’ (IPR), we videotaped the third and fifth session of 12 psychotherapy dyads, and conducted video-assisted interviews with each therapist and client separately. In total, the data material consist of 47 IPR interviews. Data were analyzed using a thematic approach.

**Results:**

The analysis process revealed two main groups. The first group consisted of dyads with a positive relational outcome, and the second group consisted of dyads with a troubled or frail relational outcome. During the initial phase of therapy, clients described feeling overwhelmed by fear and shame. Positive relational development occurred when these emotions were successfully accommodated and replaced with a growing sense of safety with the therapist. However, the relationship became troubled when the client experienced an increase in shame and/or fear during the first sessions. When forming a therapeutic relationship, it is vital that the client experience the therapist as genuine and skilled, and that the therapist is able to engage and connect deeply with the client on a person-to-person level. The article further provides a discussion on how these dyadic experiences align with the working alliance and real relationship, and how the two consolidate during the first sessions of psychotherapy.

**Conclusion:**

The current study explored the complex relational processes underlying the formation of the therapeutic relationship. Core aspects of the real relationship are prerequisites to forming a collaborative working alliance in which both therapist and client are actively engaged. Facilitating a positive relationship is crucial in the early phase of psychotherapy, and therapists can actively identify and repair ruptures at this time.

## Introduction

How can therapists cultivate and maintain the therapeutic relationship during the first five sessions of psychotherapy? What do clients find vital for a successful emerging relationship, and what relational obstacles do therapists face during the first sessions of therapy? After decades of psychotherapy research, these questions remain unanswered in the literature.

The therapeutic relationship, often referred to as the ‘working alliance’ in research, is vital for attaining successful psychotherapy outcomes (Flückiger et al., 2018; Norcross and Lambert, 2019). Not only is the working alliance a therapeutic component in itself, but a sound alliance is significantly associated with lower rates of premature dropout (Swift and Greenberg, 2012). Qualitative studies have provided insight into the relationship between alliance and dropout, with patients reporting dissatisfaction with the therapeutic alliance when asked about their reasons for dropping out of therapy, for example (Khazaie et al., 2016; Hundt et al., 2020). Other studies have shown that patients tend to rate relational aspects such as dislike of the therapist and lack of confidence in the therapist’s ability to help as important reasons for their decision to drop out (Westmacott et al., 2010). Since most dropouts occur in the initial sessions of therapy (Barrett et al., 2008), the earlier the alliance is established the better.

Moreover, problems with the working alliance have been implicated in treatment failure. Non-improved patients have described an unproductive distance in the therapeutic relationship when asked to describe their course of treatment (Werbart et al., 2015), and patients who are dissatisfied with therapy also report a poor alliance and disagreement with the therapist on how to do therapy (Nilsson et al., 2007). Moreover, patients experiencing negative or harmful therapy have attributed their treatment outcome to a failure by the therapist to engage in a caring, authentic, and collaborative manner (Bowie et al., 2016). However, a qualitative study by Radcliffe et al. (2018) provides a complex picture of the processes leading to non-response. The authors found that clients prior to and in the initial phases of therapy were overwhelmed by fears of losing control or being judged by the therapist. As a means of coping, clients actively avoided specific issues or emotions, and became compliant or passive in the face of the process. Even when disclosure felt necessary and important, it seemed impossible to open up (Radcliffe et al., 2018).

Almost 30 years ago, Gelso and Carter (1994) distinguished between three components of the therapeutic relationship: the real relationship, transference-countertransference, and the working alliance. In recent years, most research on the therapeutic relationship has been conducted under the term ‘working alliance.’ Drawing on the pioneering work of Bordin (1979), the working alliance is defined as an agreement between the therapist and the client on the goals and tasks of psychotherapy, and the presence of an emotional bond between them. Although widely used in research, this model has been criticized for being vague and open-ended (Horvath, 2018), especially when it comes to the meaning of the ‘emotional bond.’ In recent years there has been an increasing research interest in the working alliance’s sister concept, the ‘real relationship.’ The real relationship consists of two elements, realism (having a realistic perception of the other and experiencing the other as he or she truly is) and genuineness (relating to the other in an authentic way, even while each participant plays the role they must take in psychotherapy; Gelso and Kline, 2019).

Although the working alliance and the real relationship may appear similar, they have been shown to contribute differently to treatment processes and outcomes (Kivlighan et al., 2017; Gelso and Kline, 2019). Gelso and Kline (2019) point to a significant discrepancy between the two concepts by dividing the emotional bond into two sub-categories: a working bond or a person-to-person bond. They argue that the former is part of the working alliance, referring to the connection that emerges when therapeutic work is going well, the client has faith in the therapist’s ability to help, and the therapist has a fair understanding of the client’s distress. The latter, by contrast, constitutes the real relationship, and arises when two human beings encounter each other and come to appreciate and enjoy one another (Gelso and Kline, 2019). Thus, the real relationship does not relate directly to psychotherapy, but to a phenomenon common to all human relations and interactions. In the following, we will use the term ‘therapeutic relationship’ as an umbrella term for the processes underlying both the real relationship and the working alliance.

As said, much is known about the importance of the working alliance for psychotherapy outcomes. Recent studies suggest the same for the real relationship, although research is in its early infancy (see Constantino et al., 2021, for a summary). Thus, there is little doubt about the importance of establishing strong relationships early in the therapeutic endeavor. Yet, the existing research literature does not provide practical knowledge about how these early relation-building processes can be facilitated, nor how early relational barriers or ruptures can be repaired. In addition, few studies have explored how facets of the real relationship and the working alliance unfold in the first encounters.

### Study Objectives

In the present study, we seek to advance the literature by exploring how clients and therapists act dyadically to establish a therapeutic relationship during the first five sessions of psychotherapy. Using an innovative video-assisted qualitative approach, the study aims to investigate micro-processes, to provide vital insights about how the real relationship and the therapeutic alliance interconnect, and how the relationship naturally unfolds in routine care. Our goal is deepen our understanding of relational-building processes in the client, the therapist, and between them, during their first encounters.

## Materials and Methods

### Epistemological Approach and Interpersonal Process Recall

As a phenomenon, the therapeutic relationship unfolds in the rich, complex and ever-changing encounter between two people. Relational experiences are a form of implicit embodied knowledge, perhaps unnoticeable or unconscious even to the person experiencing them. To complicate matters further, relational experiences may be momentary, always shifting from 1 min to the next.

In terms of methodology, what happens within a person during the therapeutic conversation is difficult to access. In traditional qualitative interviews, the person is often interviewed in retrospect. Such ‘after the fact’ interviews may be problematic when studying relational phenomena, since the inherent micro-processes are so subtle, immediate and complex. As a result, when the session is over, a person may have forgotten about pivotal moments that occurred during the session, or may not have even been aware of them at the time.

We therefore chose the Interpersonal Process Recall (IPR) method to explore relational development at a micro-process level. IPR is a qualitative research method utilizing video or audio recordings to support the interviewee’s recollection of every moment in the session. While IPR was originally developed for supervision, the method has increasingly been used in recent years for the purpose of studying psychotherapy micro-processes (Elliott, 1986; Kleiven et al., 2020; Solstad et al., 2021a,b). In psychotherapy research, IPR typically entails video recording the therapeutic interaction; the recording is then viewed by the client and/or therapist shortly afterward, at the same time as the research interview.

Video-assisted interviews have the potential to evoke and access unspoken experiences as they were experienced in that very moment (Larsen et al., 2008), helping to identify vital interpersonal moments in the therapeutic encounter (Macaskie et al., 2015). Compared with alternative qualitative strategies, this method brings us closer to the relational phenomena we wish to investigate, enhancing in-depth exploration of experiences at a micro-process level.

### Recruitment and Data Collection

We recruited therapists from two different outpatient clinics. Clinic 1 is a public outpatient clinic located in a small city on the west coast of Norway, providing free healthcare for people with mental health disorders in the region. All of the researchers in this study are affiliated with clinic 1. Clinic 2 is a private practice with two sub-clinics (one in Bergen, the other in Oslo), specializing in intensive evidence-based psychotherapy, for charge. The reason for collecting participants from two separate clinics was that due to relatively large turnover and an extensive workload for the employees at clinic 1, it proved to be difficult and time-consuming to recruit a sufficient amount of participants from this clinic alone.

Participating therapists from both clinics agreed to invite one or two patients to be part of the study. Patient participants from the private clinic had the cost of two sessions reimbursed by the research group, in line with the principle of not having to pay to participate in research.

In total, 12 therapists recruited one patient each to the study, resulting in 24 participants across the two different clinics. Seven therapists worked at clinic 1, while the remaining five worked at clinic 2. For each patient, sessions three and five of therapy were then video recorded. We wanted to study relationship development processes within the first five sessions, but once when the therapeutic work had begun (i.e., not the first session). The main reason for this decision was based on research suggesting that relational formation within the first five sessions is vital for successful psychotherapy, particularly in terms of reducing the risk of premature dropout. We elected to study the third and fifth session to provide clients time to consider whether they wanted to participate in the study and allow the initial introductory/socialization phase of treatment pass prior to data collection. Moreover, we chose to investigate two sessions (third and fifth) to be able to follow their relational development across some time. In addition, studying both sessions enabled us to explore whether ruptures or other forms of relational barriers in session three were overcome in session five, and investigate the processes leading to either relational reparation or stagnation.

Separate IPR interviews were conducted with both the therapist and the patient within 48 h of the session. All but one participant completed both interviews, while one patient cancelled the first interview but met for the second. As a result, we completed 24 IPR interviews with 12 therapists and 23 IPR interviews with 12 patients (47 IPR interviews in total). Interviews were semi-structured, and the interviewers used a flexible interview protocol with three main questions and suggestions for open-ended explorative questions. Interviews were performed as open dialogues, to enable us to follow up on specific issues relevant for each unique dyad.

The first author conducted 31 interviews, while two clinically experienced students in psychology conducted the remaining 16 interviews as part of data collection for their final thesis. Duration of the interviews ranged from 1 h and 15 min to 2 h and 55 min, with most of them lasting for approximately 2 h. The IPR method is both comprehensive and time consuming, and as a result, we were not able to watch the entire video tape during the IPR interview.

### Participants

#### Therapists

Of 12 participating therapists, 11 were clinical psychologists and one was a medical doctor specializing in psychiatry. Nine identified as women, while three identified as men. They had between 18 months and 16 years of experience since being licensed, and were trained in various therapeutic backgrounds: emotion focused therapy (5), cognitive behavioral therapy (1), metacognitive therapy (1), and eclectic/integrative backgrounds including psychodynamic therapy and mentalization-based therapy (5). This variety in therapeutic background is representative of mental healthcare in Norway.

#### Patients

Three of the patients identified as men, while the remaining nine identified as women. They ranged in age from 24 to 50 years. The most common reasons for seeking therapy were depression, anxiety, suicidal thoughts/self-harm, and trauma/PTSD. We did not collect diagnoses as part of the data collection for pragmatic reasons. First, according to national guidelines, diagnostic assessment in public mental health services usually takes 6–12 weeks, meaning that most clients in clinic 1 would not have received their final diagnosis before the fifth session. Second, many clients in private practices such as clinic 2 do not qualify for and are not assigned a mental health diagnosis, and as a clinic they mainly worked transdiagnostically. Incorporating formal diagnoses into our analyses of micro-process relational problems did not seem beneficial beyond the already-included presenting problems, as relationship problems arise across all diagnostic categories.

### Researchers and Reflexivity

The first author is a clinical psychologist and research fellow with 5 years of clinical experience. The second author is a clinical psychologist and researcher with 13 years of clinical experience. The third author is a clinical psychologist with 7 years of clinical experience. The last author is a clinical psychologist with 15 years’ experience and a professor of clinical psychology.

Finlay (2021) defines reflexivity as the researcher’s critical self-awareness and the processes undertaken to examine and analyze own preconceptions and understandings that might influence the research. To enhance self-awareness, the interviewer wrote down immediate thoughts and reflections after each IPR-interview. In addition, the last author also listened to recordings from the first IPR-interviews and provided feedback to the interviewer. Further, the last author supervised and de-briefed with the interviewer after the IPR-interviews. Lastly, when analyzing the data, the research group strove for an open-minded and transparent analysis process.

### Data Analysis

All 47 IPR interviews were transcribed verbatim. In addition, since the IPR interviews were conducted while watching the video-taped session, we also transcribed sections from the video to add context to what was discussed in the interview. In total, this resulted in over a 1,000 pages of transcripts making the analysis process complex and time-consuming. Therefore, only the first author was able to read through the entire data material. We chose to utilize a flexible and interpretative approach to thematic analysis, as described in Finlay (2021), following the six steps of Braun and Clarke (2006). (1) The first author re-read, listened to and worked to become familiar with the data material. All transcripts were read, both separately and then dyadically, to better understand how both the therapist and the client contributed to relational dynamics and processes. Furthermore, (2) the first author generated initial codes, and (3) searched for tentative themes that represented many and various aspects of the participants’ experiences.

Thereafter, the first author prepared a full-day analysis seminar gathering the rest of the research group. The group were provided a thorough and detailed presentation of each dyad with quotes and excerpts of the data material, and preliminary themes with a frequency table providing an overview of which dyads contributed to each theme. Within the research group, (4) these themes were thoroughly discussed, revised and refined in collaboration, resulting in a final thematic structure (5). The first author then returned to the data material in order to audit the thematic structure, based on the data material as a whole, and started the write-up process (6).

### Ethical Considerations

The study protocol was submitted to the Regional Ethics Committee (REC) and approved (reference number 2015/2319). Participating therapists received two different invitation letters with consent forms, one for the therapist and one to deliver to eligible clients. The letters provided written information with contact details for the project leader, as well as the project scope and protocols, underscoring the participant’s right to withdraw at any time.

Using the IPR method in psychotherapy research warrants particular ethical considerations. First, a unique aspect of IPR is the way in which the participant becomes an observer of him- or herself, potentially inducing strong negative reactions about one’s own appearance or behavior (Larsen et al., 2008). Second, for the client, attending psychotherapy inevitably entails being in a vulnerable position, as the client is the one experiencing and displaying emotional distress and seeking help for this. It can be overwhelming enough for a person to open up to a new therapist in the first place, and in IPR the client is asked to face this process with a researcher as well. Third, the therapist is also in a vulnerable position in IPR, especially if the session does not go as planned or the therapist fails to help the client. Last, using the IPR method to study therapy dyads warrants particular attention due to the possibility that participants can identify one another in the published material. Ummel and Achille (2016) underscore that it is especially important to protect internal confidentiality in dyadic research by mitigating the risk that one participant could learn something about the other that was not intended to be shared, since there is a risk of emotional harm in research conducted with individuals who have shared an intimate experience. We were mindful of these aspects during all phases of the project, from planning, conducting interviews, analyzing data, writing and disseminating findings. Moreover, in the interview setting we strove to be open-minded, curious, accommodating and non-judgmental, and asked for feedback at the end of each interview.

## Results

When analyzing the data, we found that the dyads in this study could be divided into two main groups. The first group consists of dyads where the therapeutic relationship settled quickly, which in turn resulted in therapeutic work that both client and therapist found productive in both sessions three and five (dyads 1, 2, 3, 5, 6, 10, and 12). The second group consists of dyads with a troubled or frail therapeutic relationship, and a lack of collaborative therapeutic work (dyads 4, 7, 8, 9, and 11). Hence, the groups serve to describe two sides of relational development: the processes leading to a strong relationship (and subsequent, therapeutic work), and the processes leading to a troubled or frail relationship (and subsequent lack of therapeutic work).

**Figure 1:**
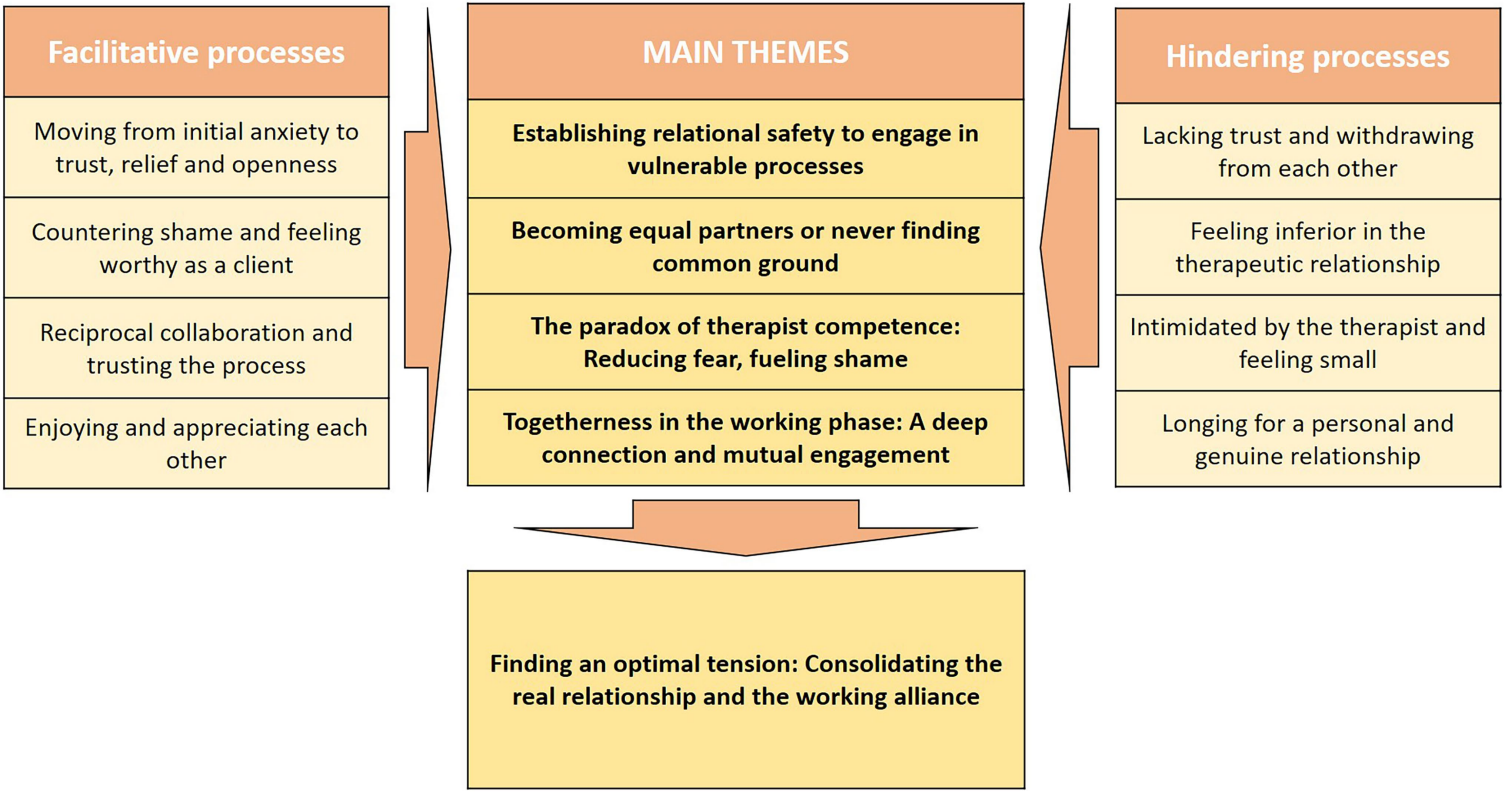
Illustration of main themes and contributing processes.

Interestingly, we found that processes that led to a strong relationship in group one were often lacking in group two, suggesting that the four themes from each group mirrored one another. We therefore settled on four main themes representing not only the processes and dynamics that supported a strong relationship but also the processes and dynamics that hindered or disturbed the relationship (see Figure 1). The first two themes cover how feelings of fear and shame (respectively) influence the emerging relationship. The other two themes cover initial relationship-hindering feelings on the part of the client, and the processes that either diminish or heighten these. We also found that a strong therapeutic relationship appeared to co-occur with the initiation of therapeutic work. This consolidation of relational processes and technical aspects of therapy is described in the fifth theme.

In the following, we detail each of the four themes, starting with the experiences and processes underlying a positive relational outcome (group one), followed by the experiences and processes underlying a troubled relational outcome (group two). The discussion of each theme includes a dyadic dialogue, aiming to illustrate how both client and therapist contribute to the process. An overview of which dyads that provide information to the different themes is provided in Table 1.

**Table 1:** Representativeness table.

Themes/Dyads	1	2	3	4	5	6	7	8	9	10	11	12
1. Safety/withdrawal	x	x	x	x	x	x	x	x	x	x	x	x
2. Equal partners	x	x	x	x	x	x	x	x	x	x		x
3. Competence	x	x	x	x	x	x	x	x	x	x		x
4. Togetherness	x	x	x	x	x	x	x	x	x	x	x	x
5. Optimal level of tension	x	x	x		x		x	x	x	x		x

Table illustrating which dyads that provided information to each theme. The colored columns represent dyads with a troubled or frail relational outcome, while the uncolored represent dyads with a positive relational outcome.

### Establishing Relational Safety to Engage in Vulnerable Processes

All clients described starting therapy as an anxiety-evoking experience, and remembered feeling fearful or apprehensive particularly before the first session. These feelings were often tied to concerns about who the therapist was as a person and whether he or she was interested or skilled enough to help them. For clients in the group with a good relational outcome, these feelings quickly became tolerable during the first five sessions of therapy, making room for the therapeutic work to begin. However, for clients in the group with a troubled or frail relational outcome, fearfulness became overwhelming and was highly present during both the third and fifth sessions, propelling feelings of despair and helplessness in both client and therapist.

In the positive outcome group, the therapists described being mindful of clients’ initial fears and anxiousness, and worked consciously to make clients feel safe about therapy. They usually described safety as foundational, and said they believed that if fear was not dealt with, it could compromise the therapeutic process. In some cases, it was challenging to establish safety, but the proximity it could yield made it worth the effort. These therapists described how they systematically worked to end fear using the presence of emotions and the client’s emotional expression as a compass:

‘Right here, I experience that he is in a mode where he feels safe with me, actually. He wipes his tears, but also lets them run a bit before he wipes them. And… I kind of feel closer to him now. As if we are closer to one another, since he is letting himself be vulnerable and in touch with what is painful to him’ (therapist, dyad 5).

From the corresponding client perspective, clients in group one described a process where their fearfulness diminished as therapy progressed, which awakened interest and engagement as a result of growing trust in the therapist. The following quote illustrates this:

Interviewer: ‘So, how is it to… sit there, waiting for the session to begin?’

Client: ‘I guess there is fear. Yeah. Fear of collapsing emotionally. […] But there is also an… excitement or curiosity about what’s to come. Which originates from the trust I feel with her’ (client, dyad 10).

As the relationship began to be cultivated, clients described a process of gradually feeling safe. Words used to describe this process were: relaxed, relieved, calm, comforted and pleased. When watching their sessions, clients typically pointed out their sudden change in posture (leaning back, a softer facial expression, and a relaxed and open sitting position). As mentioned, feeling safe was often accompanied by increasing mutual engagement, manifested by the client becoming more talkative, laughing or smiling, but also the opposite, in the form of more tears, grief or despair.

Hence, feeling safe took many forms, but in common for all clients, the experience of safety was fundamental. Some clients believed feeling safe with the therapist to be the most important thing in therapy, as previous relationships in their life had been destructive, violent or dangerous. One client pointed to a moment in therapy when she particularly felt safe and explained: “[Being able to cry] means that I feel safe. I can be sad around her. And when I first cried, I experienced that it was helpful sharing the things I’ve kept to myself for so long. Really painful things” (client, dyad 12). Hence, feeling safer helped the client to let go and share more of their story or inner experience, spiraling into new moments of relational encounter and deepening their feeling of connection.

However, although most clients described this process vividly, not all dyads successfully resolved initial fears and apprehension. This was particularly the case for dyad 11, and was also present in dyads 4, 7 and 9. These dyads were characterized by client withdrawal, sometimes in the form of being unfocused or frequently shifting focus to unrelated topics. At other times the client receded into the background of therapy as a passive, non-responding observer. In dyad 11, the client described not feeling safe enough to open up. She explained, ‘I automatically distance myself, it is hard not to do it,’ and attributed this tendency to the need to protect herself in past destructive relationships.

Having been in therapy for a couple of years with a different therapist, she recalled that time was necessary for her to feel safe: ‘It gets easier with time, but essentially I just need to decide to be more open’ (client, dyad 11). Yet, she also reflected on the differences between her past and current therapists, recalling that her previous therapist was easier to read, which helped her relax: ‘It felt so real and genuine, kind of. She threw herself back in the chair, breathed deeply and just… everything. Yeah, those things’ (client, dyad 11). Although she understood that distancing herself from the therapist made it difficult for her current therapist to help her, she described being unable to let go of her fears, to trust her new therapist and engage collaboratively. She was unsure of what held her back, but subtly suggested that she missed the aliveness, genuineness and expressiveness of her previous therapist. Knowing that it is also up to her to form a relationship, she described beginning a process of gradually deconstructing the wall between her and the therapist, although she was not there yet.

In the meantime, for the therapist, this was a draining dynamic. The therapist felt that she went out of her way for her client but got nothing back: ‘Frankly, she [the client] does nothing for our relationship’ (therapist, dyad 11). Both sessions stood still, and client and therapist did not really accomplish anything significant together. The therapist described feeling exhausted, disengaged and irritated, both at herself and the client. The lack of involvement on both sides led to a mutual (unresolved, never communicated) resignation in session five.

Accordingly, finding common ground to do the therapeutic work was not solely the therapist’s task. The therapist had to lay the foundation, helping the client feel safe enough to engage. Thereafter, at some point, clients recalled taking a leap of faith, deciding to go ‘all in.’ Most clients, in some way, described a similar process, of gradually letting their guard down and engaging in therapy.

### Becoming Equal Partners or Never Finding Common Ground

In addition to fearfulness and apprehension, feelings of shame were also present during the initial therapy sessions. While feelings of apprehension were described more somatically (e.g., nervousness, tight or closed-off body language), clients described shame as a feeling of not deserving therapy, feeling small and stupid, or feeling inferior in the therapeutic relationship.

When watching their sessions on tape, several clients were surprised to see themselves clearly displaying signs of being uncomfortable. It was striking for them how visible their body language was, and they pointed out their tendency to look down, avoiding eye contact or taking a submissive body posture. One client noticed her own body language while watching her session, commenting that she tended to gaze down instead of meeting her therapist’s eyes:

‘I guess I do not want to be in contact with all of this… It is very strange for me, and there is probably a lot of shame and guilt and all the things you look down for… And, it is distressing and perplexing. I do not know why, I see that I often gaze down, but usually I’m a person who looks a person in the eye when talking’ (client, dyad 10).

Despite its visible nature, shame was not addressed explicitly in the session. However, many patients felt that their therapists dealt with it indirectly through being caring, kind and accepting. One client commented:

‘When I’m sitting there, I’m looking at that chair [behind the therapist]. I rarely meet her eye, not sure why… I guess it is because what we are talking about is so embarrassing. However, I see that she’s really focused on me […] and I can choose to look at her when I’m ready’ (client, dyad 5).

From the therapists’ point of view, working to decrease shame was explicitly on the agenda for the initial sessions. In practice, this meant that they put aside technical aspects of therapy and focused on a real human-to-human encounter. One therapist explained: ‘I want to show [the client] that you are not weak or stupid, and there is nothing wrong with you. […] Right there I believe I am conscious of our brand-new relationship, so I would rather be on her team right away, decreasing the shame, and take a more challenging stance later on’ (therapist, dyad 7). Another therapist described: ‘And… this sounds really silly, but I find it important to try to really see her in the middle of all this… give her a sense of me being really interested in trying to understand her, helping her’ (therapist, dyad 2).

Other ways of showing acceptance to decrease feelings of shame and unworthiness included deliberately remembering important details, such as names of the client’s spouse, family or friends, critical events or bringing up themes from past sessions. Moreover, being genuinely curious, accepting and present helped therapists to understand the client’s situation: ‘In this moment, I believe I am following her very closely. I repeat her words, expand them a little, creating an experience of being understood, and of closeness’ (therapist, dyad 7). Therapists also talked about a genuine and real care for the client as a person worthy of therapy, and these feelings were deeply personal: ‘As a human, I feel love for her. I feel like: oh, I like her’ (therapist, dyad 9).

Successful resolution of initial shame gradually led to a sense of equality and collaborative partnership for the dyads with a good relational outcome. One client put it this way: ‘The difference between me and him is not as big, at least that’s how I feel. […] I feel as if I am talking to someone I know’ (client, dyad 6). He appreciated that the therapist made room to talk about their common interest in television shows, and described how this interaction, as two casual human beings, subsequently enabled therapeutic work: ‘Frankly, our relationship is a lot better after this session than before. […] It changed my mindset, our dynamic. Actually, we have accomplished so much, in what I view as such a short time’ (client, dyad 6). However, in the remaining dyads, shame recurred several times during the initial sessions, and for some clients it colored their experience of therapy. Sometimes it led to withdrawal and resignation from the therapeutic relationship; at other times it could result in a direct confrontational rupture, in which the client clearly displayed anger or irritation toward the therapist.

In dyad 4, the client described fearing that his therapist would reject him if he revealed his true self, and that he needed to maintain her positive impression of him, meaning that he could not be honest with her:

Client: ‘[I am afraid I will] ruin her positive impression of me… […S]ince she sees me as a goodhearted person, then I have to be one.’

Interviewer: ‘And that’s scary…’

Client: ‘Yeah, it really is. It is a scary situation’ (client, dyad 4).

More generally, both sessions in dyad 4 were characterized by moments when the client did not feel correctly understood by his therapist. Indeed, at some points, the therapist did explicitly misunderstand him, and usually he took this on himself, claiming that he was a difficult man to understand due to his accent or personality. When he told the therapist about his suicidal thoughts, she did not respond empathically, but rather took notes and listened. When the interviewer asked him how it felt to tell the therapist this, he replied: ‘I find it embarrassing to talk about these things [suicidal thoughts]. I am a grown man of almost fifty, and should not have thoughts like this’ (client, dyad 4). Later he said:

‘She does not confront or respond to what I just told her… She moves on. The last thing I said was “I guess it is normal,” and then she replies instead to that. And the other things I said, I do not think they moved her… so she does not go deeper into it’ (client, dyad 4).

He felt left alone with his feelings of shame and guilt, as his emotions never reach the surface. He seemed untouched by his constant suicidal thoughts, but admitted that he really need help with handling them. Yet, when the therapist attempted to steer the conversation into a therapeutic project, he lost his concentration and did not catch what she was saying:

‘I struggle a bit with concentration. So when the therapist talks for too long, I lose it [concentration]… and then it is very tempting just to pretend to understand, saying yes or no out of courtesy. I struggle a bit with that’ (client, dyad 4).

Interestingly, both client and therapist experienced that the other was talking a lot and dominating the session. From the therapist’s perspective, she felt that she had to break into the client’s monologue to have a say. Generally, she finds his suicidal thoughts difficult to handle and struggles with finding a balance between letting him talk about this versus not taking responsibility for his life. She worries that if she lets this through, his suicidality will take over and become the main theme of the sessions, and she does not want that:

‘What he is saying is pretty serious. A plan for how to commit suicide. So it is pretty serious. But I did not go further into it because… our deal is that he will tell me if the suicidal thoughts increase, he will let me know. That we should not spend our time talking about… suicide and those things’ (therapist, dyad 4).

The interviewer asked how this may have affected the relationship between her and the client, and she answered: ‘I do not know’ (therapist, dyad 4). However, she did notice that the client lost his concentration during her longer utterances, which she took into consideration. Yet, this made it challenging to establish a therapeutic focus, as her attempts to socialize him into a therapeutic model resulted in him losing concentration. After session five, both of them described the experience of lacking a joint therapeutic project. The therapist remained unaware of her client’s feelings of shame, embarrassment and inferiority; they never become equal partners.

Dyad 9 also ran into problems. After the third session, the client stated in the interview that she felt guilty for exposing her family in therapy. She worries that she presented her parents in a bad light and that the therapist has a mistaken impression of them. In general, she felt that her therapist did not really understand her or her needs, and kept pushing to dig into the client’s past while the client wanted to focus on the present: ‘Hm…. Well… Eh… I guess sometimes I felt that maybe [clears throat] [the therapist] wanted to go somewhere I did not want to go’ (client, dyad 9).

The client began the fifth session by talking about her relationship with her boyfriend, and felt rejected as the therapist cut her off and directed her into another topic:

‘And maybe I also reacted in this session when – since I want to focus on the relationship and that is the most important thing for me now, but at one point she said: “Well, let us put him to the side…” and then I thought – felt: “Oh… but this is what I feel is important. This is the main reason I came in the first place”’ (client, dyad 9).

The client struggled with knowing how to handle the situation; at first she got agitated and unfocused, jumping from one topic to another: ‘And in some ways, it felt as if I was talking to anyone, just listing whatever had happened lately […] Well, maybe I start jumping from topic to topic as a way of trying to find something that feels meaningful’. Later in the session, she described giving up and becoming passive:

‘Yeah, I did not feel that I agreed with her there and then, and after that I wasn’t really interested in working on something else either […] Yes, I just sit there listening, waiting for something significant to come up. […] But eh… kind of… it is difficult to kind of… take control… or whatever [laughs] This is just all wrong for me’ (client, dyad 9).

The client felt that she was in no position to steer therapy, as she herself was not a psychologist: ‘But um… I am very unsure whether I know what is best for me’ (client, dyad 9). Instead of voicing her opinions and needs, she became submissive and let herself be overruled, with the consequence that she felt guilty about what she said and regretted not standing up for herself when the session was over. From a dyadic perspective however, this was not an easy process for the therapist either. She described feeling impatient and struggling to establish a therapeutic focus:

‘What I am struggling with this session is… how can I help her give herself permission [to explore her emotions] without being too pushy? How much can she take before… her defense or… armor comes back on? Because [the armor] is very on and off during the session, at least that is my experience of it’ (therapist, dyad 9).

She wanted the client to focus on herself and her own difficulties, and not put her boyfriend’s needs ahead of her own. She (the therapist) described the therapy as a duel—a constant combat where her attempts to empathically understand the client and take her side were rejected—and she felt that she failed to establish a therapeutic focus. Yet, despite all the challenges, she described having warm and affectionate feelings for her client. Reviewing a positive moment in therapy, she elaborated:

‘The fact that she’s both showing and seeking… showing vulnerability, and seeking comfort, or acknowledgment from me. That is something that warms my heart. It is good for me to see that this is a safe place for her and that she allows herself to show her feelings. So this feels good for me, and I want to give her love and caring. That is my feelings in the moment. And then, when I sit here [during the interview], I am sitting here with a giant self-critic thinking: “Darn it, why did not I bother to contain it better?”’ (therapist, dyad 9).

Later in the interview, she reflected on how she kept her client at arm’s length: ‘Not that close that it becomes “me and you,” kind of. That I… keep her at a distance’ (therapist, dyad 9). She felt the need to keep a distance between herself and the client, which she believed was a result of her own wounds. She wanted the therapy to progress, impatiently, and describes feeling obliged to push for change; she wanted them to reach the finish line as fast as possible. At the same time, she felt that she was failing as a therapist and described being highly self-critical. Shame was present for both therapist and client. As a result, both of them end up struggling in silence, neither of them aware of the other’s thoughts and feelings. This illustrates the characteristics of dyads which did not achieve equal partnership, despite high motivation on both sides. The sessions were colored by feelings of guilt and shame, from which no one knew the way out.

### The Paradox of Therapist Competence: Reducing Fear, Fueling Shame

All clients explicitly underscored the importance of perceiving their therapist as knowledgeable, wise and skilled. In fact, it was crucial to them to have trust in the therapist’s ability to help them. This theme encompasses, for example: the therapist providing new insights and understandings, instilling hope, executing successful interventions, knowing what to do, having a plan, and the client experiencing the therapy as useful overall.

In the group with positive relational outcomes, clients described it as necessary to give up control in order to make therapy effective. Knowing that their therapist had seen and heard similar things before and knew what to do enabled them to let go and engage fully:

‘I feel that she’s… in control. She knows what to say, making it feel… not less uncomfortable, but more safe. It is uncomfortable and no fun at all, actually quite horrible, but also okay, in a way’ (client, dyad 2).

The following quote illustrates this further: ‘She is not stumbling, very safe and very steady in her work. Everything seems thought through, and she has a plan. She is very skilled in the method she is working with’ (client, dyad 5). This client further explained how this helped him to relax and engage in their therapeutic project, which entailed evoking painful emotions. From the therapist’s perspective, displaying skills and knowledge was important to help the client feel safe. The therapist reflected on the same process in her interview: ‘I hope to create a sense of safety for him as result of me knowing what we are doing and where we are heading’ (therapist, dyad 5).

Furthermore, clients appreciated a therapist who responded fast and intuitively, without hesitating. Experiencing the therapist as skilled, trained and wise created a trusting therapy environment, in which the client could relax and engage. However, sometimes therapists were over-eager in their application of therapeutic interventions, creating stressful or hasty therapy conditions: ‘Right there… […] Maybe I needed a break, to think a little, maybe it all went too fast’ (client, dyad 5). At other times, as detailed above, clients felt overruled when the therapist focused overly on interventions or sharing their own interpretations.

Thus, although clients underscored the importance of getting something out of the session, competence could also backfire. One client put it this way: ‘I am particularly pleased whenever he brings forth a new understanding, one I can use in my life to make it better’. On the other hand, she continues: ‘I fall off as soon as he starts explaining things I already know, since I’m nurse, working with people myself’ (client, dyad 8). She elaborates: ‘You kind of feel… small and stupid. Like, he is thinking he must explain things very carefully, or else I will not understand’. She further stated that she wanted something more concrete, some tools or methods. In this particular moment, she did not follow the therapist in his attempt to explain her problems and how to solve them. This proceeded into a confrontational rupture, where she directly expressed anger toward her therapist.

Therapists, however, felt obliged to provide effective and accountable care. They carry the vast responsibility of an effective and helpful course of therapy and often found themselves being dragged between the client’s needs, on the one hand, and the demands of producing a good outcome, on the other. The following quote serves to illustrate this:

‘Right here I was thinking we have to write down three goals… Eh… Announcing: now we are making a plan! [clapping her hands] Come on! [laughs]

(Interviewer: What is that all about?)

Eh… [whispers] the system? And of course, I want to know that we are heading somewhere, and that we have something to work on. I am very much afraid that they keep coming here and nothing happens. […] I am afraid of not being useful to them’ (therapist, dyad 12).

There was a sense of rush in all dyads, regardless of whether the therapist was working in a public outpatient clinic or in private practice. In both situations, therapists were highly concerned about the effectiveness of their therapy and invested time, money and energy into becoming better and more skilled. In our data, most relational ruptures seemed to occur when the client felt pushed into something they were not ready for or did not want, and although therapists felt an urge to be effective, it rarely yielded positive results. On the contrary, in the sessions with high intensity and reciprocal subjective experience of progress, therapists were glad they were patient with their clients, as illustrated here:

‘At the end of this session, when we summarize things, and – where I truly felt this good feeling that, “you and me, we can take our time,” kind of. Where I can tolerate doing things at her pace, without feeling that I am doing a bad job’ (therapist, dyad 10).

Evidently, clients responded differently to the therapist’s use of competence, and clients’ accounts illustrate how displaying skills and knowledge comes with a catch, requiring thoughtful and careful handling. On the one hand, competence could extinguish fear for those clients who initially feared they could not be helped. On the other, it could fuel shame in cases where the client appeared to be burdened by shame and feelings of inferiority.

In these cases, healing inequalities in the relationship seems to be key, as the person-to-person relationship needs to be restored before they can keep on working. Slowing down, being gentle and humble as a therapist, offering small breaks when dealing with something difficult, following the client’s lead and intervening on the client’s terms, were all important. Altogether, clients appreciated when therapists found a balance between leading and following—in other words, being a skilled professional in a collaborative and attentive way, without compromising the (real) relationship.

### Togetherness in the Working Phase: Experiencing a Deep Connection and Mutual Engagement

The most frequent theme throughout the interviews was that of a growing sense of togetherness being vital for the process of building a strong therapeutic relationship – mentioned by every single client in this study. This encompasses therapist behaviors and attitudes such as genuineness, immediacy, self-disclosure, shared humor and intense, uninterrupted focus on the client as a person. In specific facilitative moments, clients tended to comment on the therapist’s presence, engagement and immediacy. Common descriptors used in relation to the therapist were: active, engaged, exclusively focused on them, genuine, and real. Here is an example:

‘She is very herself, in a way. And, I like that [smiles]. She does not take on a solely professional role, despite the fact that she is a psychologist. And the way she brings herself into the therapy room makes it so much easier for me to be myself too’ (client, dyad 3).

From the therapist point of view, most therapists experienced it being easy for them to establish togetherness in the relationship, as this required human qualities coming naturally to them. Here, therapists mentioned genuineness, compassion, empathy, patience, congruence, and presence as important relational facilitators. Sometimes, positive feelings toward the client came naturally. At other times, it required some work. One therapist explained that her client is not the type of person she usually empathizes with, as the client represents something that was challenging in her own childhood. She consciously has to work to be empathetic:

‘How I do it? When I notice that this just does not move me as easily as it could, right. When I notice that I – I both feel your pain but simultaneously, I get a bit annoyed and impatient. That is often my signal, that I am becoming impatient, which I know does a lot to my empathy. So, the first thing I do… I physically lean forward. Coming closer. Inviting contact. And when she leans toward me, I kind of feel… trust. And the more trust I feel, the more responsibility I take’ (therapist, dyad 10).

However, she also knew that empathy could land differently if it became too much or inauthentic:

‘I believe one of my strengths as a therapist is that I can – or that I am warm. That clients experience me as warm. And that has been more challenging with this client than with others. […] I experience that she has a form of self-criticism that is so… strong, that sometimes she kind of gives me two different tasks: it is good to receive warmth, but then all of a sudden… that warmth becomes a little offensive. […] And I know this is a risk of my kind of empathy, that I can become [laughs a little] a bit too *–* or where it becomes evocative, like I pity them, making her feel weak” (therapist, dyad 10).

The client in this dyad confirmed that she felt lots of shame as she began therapy: ‘I find it embarrassing, being in therapy. I do not want to tell anybody. So with that in mind, it has been a *very* good experience for me’ (client, dyad 10). She continued: ‘Personally I believe most of the energy that… radiates from her, has to do with what kind of person she is’ (client, dyad 10). Further, it was significant for her in moments when her therapist leaned forward and welcomed her tears and pain. She described her therapist as fully present and there for her. At the same time, she experienced the therapist as relaxed, genuine and open. The client commented on her casual, almost slouching sitting posture, standing in marked contrast to her own tightness and attempts to ‘hold it all together’ – ‘showing me that I am allowed to be more relaxed than I am used to’ (client, dyad 10). Further, the client appreciated how her therapist invited proximity and connection:

‘I noticed that she was sitting close, and it might have been to connect with me, since I actually could not make eye contact. She does not know me very well, but she sees [laughs] quite clearly that I, um… She sees that I… am not there. Not really present. I’m looking away, I’m looking everywhere else. […] My understanding is that she is trying to engage me, remind me that “I’m here”. Get closer somehow, because I am pulling away’ (client, dyad 10).

The client and therapist here found common ground, relating to each other as human beings, not roles. Time and again, clients valued authentic moments in therapy, where the person of the therapist came into the foreground and they met as equals. Shared sense of humor played an important part here, and clients described sharing moments of joy as a major contributor to the feeling of togetherness. Not only was it a big relief to laugh together for the first time, it also added a sense of uniqueness to their relationship. Some clients mentioned that laughing with the therapist added some light to the darkness they were exploring together:

‘The fact that we are able to talk and mess around, that it is not super-serious all the time… […] That we are having an actual conversation, that’s positive. […] Especially since we are talking about what bothers me, that we are able to pull something positive into the conversation, having a good time together while talking about it. Making it all less heavy and gloomy’ (client, dyad 6).

Furthermore, for clients, togetherness was rooted in perceiving the therapist as genuinely caring for them, no matter what they brought to therapy. For some, this was a new but longed-for experience:

‘It is so strange to be not-fine, but still feel so taken care of. To be allowed to open up around all that difficult stuff, and that is okay. It is very rare in life to feel okay about not being okay. So that is… that is very special’ (client, dyad 8).

All clients described their therapist as warm, welcoming, caring and accommodating, which in turn repaired an initial sense of inequality in their relationship. This helped clients feel safe and relaxed, worthy and equal, but also deeply connected in the sense of being a team, working together to solve whatever problems they had. Hence, feeling deeply understood, acknowledged and accepted as a person was the safest route out of feelings of shame and unworthiness. This way of being together went beyond the technical aspects of therapy, but simultaneously made room for real therapeutic work, as it made the client ready to engage.

However, despite the fact that clients perceived togetherness as exclusively positive for their therapeutic experience, many therapists feared that a major focus on relational aspects could compromise an effective therapeutic course. They explained that although they were able to connect deeply with their clients, they had experienced times when therapy stood still. As one highly experienced therapist put it: ‘Having a good relationship is not enough, the patient needs to experience progress as well’ (therapist, dyad 9).

### Finding an Optimal Level of Tension: Consolidating the Real Relationship and the Working Alliance

In the group with positive relational outcomes, it was clear that there was already ongoing therapeutic work by session three, in the form of characteristic interventions (chair work in EFT therapies, exposure therapy in CBT), presence of emotions (usually the client crying, feeling angry or displaying other emotions), and general high intensity (frequent therapeutic interventions, fluctuating emotions, reciprocal engagement). In these dyads, client and therapist were working seamlessly together to solve an explicit problem and experienced progress from sessions three to five.

The former themes illustrate how initial feelings of apprehension (shame and fear) were resolved through togetherness and thoughtful use of therapist competence. Successful resolution manifested as a growing sense of safety, relaxation and openness in the client. One client summarized the process as follows:

‘The take-home message is perhaps… um… safety, and caring and empathy, that’s one bit. And then the feeling that the therapist has lots of experience, training and competence. And when those combine, things turn out very, very well. Because I’ve been surprised and struck by how quickly things… how far we have gone and how fast the connection… or safety was established. How fast we were able to broach difficult subjects. Um, like already in the second session, I am already feeling better?! And these things only happen in safe and sound places’ (client, dyad 5).

A significant finding in the successful relational outcome group was that a strong relationship was not necessarily a smooth one. On the contrary, these clients stressed that a good therapeutic process entailed evoking painful emotions and being (fairly) challenged. One client recounted how she was struggling to hold back her emotions while on her way to the therapist’s office. As soon as she entered the therapy room, her therapist gave her a friendly greeting and she burst into tears. As she did so, the therapist worked to expand her grief, pushing her deeper into despair and emotional pain. In the interview, the client elaborates:

Client: ‘And when she [the therapist] says: “It is almost as though your body needs to cry, letting it out,” she breaks my heart! [cries] Because it is so true.’

Interviewer: ‘How does that feel?’

Client: ‘Painful. I am in indescribable pain. Yet, it feels so right’ (client, dyad 10).

For clients, the fact that they were sharing difficult emotions in therapy indicated that they were working with something significant and important. Other clients reflected that being challenged could result in relational growth. This was particularly present in dyad 8, in which the therapist carefully suggests working with the client’s difficulties in close relationships:

‘He is now touching some aspects of my personality and my life […] which I’ve always known were there, but have never been able to acknowledge to myself. I may not be there yet, but I am getting there. We are getting there. To be honest, being scared has never felt this safe’ (client, dyad 8).

For therapists, it was a demanding task to move from building a personal relationship to engaging in a work-based instrumental alliance. Almost all therapists found themselves in a situation where they felt on thin ice, but still had to ‘take a deep breath’ and sacrifice the relationship to make progress. Sometimes this transition went well, while at other times it destabilized the foundational relationship. It is worth noting that the most common process contributing to the failed alliance in the group with troubled relational outcomes was the therapist pushing too hard with therapeutic interventions, resulting in resignation/withdrawal or direct confrontation from the client. In dyad 10, the therapist concluded that it paid off to be patient with her own agenda, since as soon as the client was ready, the therapeutic work started automatically and naturally:

‘And that’s the fun part, when you realize that the things you thought caused a bad process were actually important – maybe even necessary to get anywhere, and that really helped you understand more of the client. […] Yeah, not rushing into being productive from the start. […] I have to be a bit careful that my aim to be a productive therapist must not be at the expense of her experience of being … a client who is “good enough” [slight laugh], if I can put it like that. I do think there is a risk that she might feel that way’ (therapist, dyad 10).

Furthermore, having some tension in the relationship was also important for therapists, who described being guided by emotions and connection. When difficult emotions emerged, they knew they were on the right track, working with something significant. It was often described as a big relief when a client suddenly started crying or came into contact with difficult emotions or memories in other ways. Often, it required a lot of work to get there, and a powerful tool was for the therapist to evoke emotions within themselves (actively picturing what the client was experiencing, putting themselves into the client’s situation and trying to make sense of their own feelings). One therapist explains:

‘Yeah, just to connect to her. To really put myself in her shoes. When she starts talking, I picture the little child and the situation she’s in, and how it feels to be a mother who needs to protect her child. It almost makes me want to cry myself’ (therapist, dyad 12).

Establishing a collaborative *working* alliance therefore relied on a strong real relationship—otherwise the use of interventions could threaten or destabilize the emerging relationship. In other words, the relationship had to come first, and the alliance second. In togetherness, client and therapist became allies, working together to solve problems. The therapist could become a helper without the client feeling helpless; the client could be vulnerable without feeling weak; and they met as equals despite their different roles and positions in the relationship.

## Discussion

During the initial sessions of psychotherapy, we found that therapists have to maneuver within a complicated relational landscape, with two (sometimes conflicting) tasks at hand: cultivating and maintaining a real relationship and establishing a collaborative working alliance. Furthermore, we found that starting therapy elicits difficult emotions in the client, particularly fear and shame. How these feelings are initially dealt with impacts both the emerging real relationship and the working alliance. The data revealed two main groups. The first consisted of dyads where the relationship and the alliance developed smoothly, with a naturally occurring transition from getting to know one another as genuine people, beyond their roles as client and therapist (the real relationship), to a work-based therapeutic alliance in which both are actively engaged. In this group, initial fear and shame gradually resolved, replaced with trust and mutual engagement. In the second group, the relationship was brittle, frail or unstable. Here, feelings of shame and/or fear became overwhelming for clients, hindering their involvement in therapy and the therapist’s ability to initiate a constructive therapeutic process. In some cases, this dynamic appeared regularly throughout both the third and fifth sessions, propelling despair and helplessness in both client and therapist.

### Disentangling the Alliance-Change Knot

In this study, we found that the most robust characteristic of a therapist- and client-defined strong relationship was the presence of therapeutic work, suggesting that the two are closely connected. In psychotherapy research, it is difficult to distinguish between relational and technical variables: are positive alliance ratings the result of effective treatment methods, or is effective treatment reliant on a good alliance? A recent meta-analysis by Flückiger et al. (2020) has shed light on this debate, showing how early alliance and symptom improvement actually go hand in hand, interwoven in a reciprocal and dialectical manner. In other words, it is as though there is a positive upward spiral of greater alliance and greater improvement/fewer symptoms. Drawing on this insight, the current study illuminates *how* the two interact from a lived first-person and dyadic perspective.

First, we found that it is key for clients to perceive their therapist as skilled, knowledgeable, and having a plan of how to conduct therapy—meaning that the personal bond by itself is not enough to form a strong relationship. Thus, the client needs to feel assured that the therapy will yield an actual positive difference in his or her life, in line with findings from other qualitative research (Binder et al., 2009; Lavik et al., 2018). Perhaps, from the client perspective, there is no clear-cut difference between treatment method and relationship. Rather, they co-exist, mutually affecting each other. In other words, a strong relationship, from the client perspective, entails trusting in an effective treatment method.

Second, bringing forth a dyadic perspective, therapists usually experienced the working alliance as strong when there was, simultaneously, ongoing therapeutic work. Their experience was that when the real relationship was established—manifested in trust and faith in the therapeutic project—therapeutic work began seamlessly, spiraling into an emerging collaborative working alliance. On the other hand, when the real relationship was frail, there was less room for therapeutic work. In these situations, therapists tended to feel paralyzed and confused.

Third, when the real relationship started to develop, it manifested as a transition from initial fear and shame to a growing sense of safety within the client. Furthermore, we observed that safety was fundamental, serving as a ‘launching pad’ for self-disclosure, openness, and active engagement (the working alliance). This transition within the client is vital for the success of therapy; research has repeatedly demonstrated that the client themselves is the most potent change factor in psychotherapy (Bohart and Tallman, 2010). Nonetheless, given that contemporary psychotherapies (which are increasingly intensive, goal-directed, emotion-focused and brief) require a great deal of effort from clients, finding ways to mobilize the client in initial sessions is crucial. Accordingly, a recent psychotherapy process study by Negri et al. (2019) investigated the client’s linguistic style in the first session of therapy. They found if clients took time to talk about themselves, focusing less on general somatic symptoms, and described their emotions and affects in a non-neutral way, then the therapeutic alliance tended to be stronger. In other words, a good alliance appeared to allow the patient to come into contact with their inner world, and starts a process of joint emotional elaboration (Negri et al., 2019).

Following this line of reasoning, our findings support the notion that treatment method and therapeutic relationship are inseparable. Furthermore, our findings also suggest that there appear to be two phases in which a therapeutic relationship emerges: (1) the relational processes that occur when a person encounters another person (real relationship), and (2) the relational processes that occur when the dyad takes on a therapeutic mission (the working alliance). A strong therapeutic relationship can be seen as a successful consolidation of both.

### A Dyadic Perspective on Relationship Formation

Normal human development relies on the cultivation of relationships with others, making us hardwired for connection and relationships from infancy. No wonder clients find the therapeutic relationship the most helpful aspect of therapy (Binder et al., 2009; Norcross and Lambert, 2019), consistent with the fact that moments of togetherness were the most appreciated and frequently mentioned by the clients in this study. Although the therapeutic relationship is the most studied variable in psychotherapy research, the concept as we know it has been developed by researchers, and less research has been devoted to understanding the relationship from a client or therapist perspective (Krause et al., 2011). It seems logical that clients, therapists, and researchers all perceive the therapeutic relationship from different angles. Each perspective will yield different, but valuable, information about what the relationship is, could be, and how it naturally enfolds. This warrants the question: how does the concept of the therapeutic relationship align with the dyadic experience of clients and therapists?

Compared with therapists, clients put greater emphasis on embodied aspects of the relationship (feeling relaxed, safe and engaged). The relationship was something *felt,* at first in terms of feeling less ashamed or anxious, which in turn made room for who they were as a person. Feeling safe arose from multiple moments in which the client experienced the therapist as genuinely caring and friendly, but also professional and skilled. Our results imply that for clients, the therapeutic relationship is something real, intimate and unique which they share with the therapist, as much manifested in moments of joy and common humor as in moments of togetherness in vulnerability and despair. The relationship becomes a space for clients to reveal who they are, and to evolve and grow into something new. Hence, if we were to define the therapeutic relationship from the accounts of clients, it always came back to feeling safe and relaxed with the therapist. However, these aspects are not embedded in the traditional concept of the working alliance. Rather, they resemble core aspects of the real relationship (Gelso and Kline, 2019).

In contrast to clients, some therapists were concerned that being overly focused on the relationship alone could result in lack of therapeutic progress. Some had had the experience that their willingness to maintain a relationship above all else had left therapeutic work stagnant. Therefore, establishing a working alliance on top of a real relationship was crucial. Although the research literature considers the working alliance and the real relationship as separate constructs (Gelso and Kline, 2019), we found that both clients and therapists experience them as dimensional, as phases on a continuum: the real relationship needs to be established first, and then a collaborative working alliance can be formed. For clients, encountering the person of the therapist counteracts initial feelings of shame or inferiority, while a growing trust in the therapist’s professional skill resolves fears about being unhelpable. Therapeutic work begins when the client feel safe. Therapists, however, work consciously to make this transition happen and described it as deeply satisfying and meaningful to engage in a collaborative working alliance. They themselves were driven by the emotions and connection that came when the client started to open up. For therapists, being able to help and relate to clients in a constructive way was what made it worthwhile. This is in line with recent dyadic qualitative studies, showing that technique and relationship are not separate constructs, and that the therapist combines their authentic, personal and professional parts to form a relationship that is both real and therapeutic (Bernhardt et al., 2021; Råbu and Moltu, 2021).

### A Lost Space: Lessons Learned From the Group With Troubled Relational Outcome

However, not all dyads successfully established a therapeutic relationship by session five. What can be learned from the dyads in the troubled relational group? First, we found that a frail relationship severely disrupts therapy, making it impossible to engage in a constructive process. In dyads 4 and 7, particularly, the therapist’s response to the lack of mutual engagement was to take a leading role and push forward a therapeutic project that was not the client’s. This proved to be fatal for the relationship.

The research literature on rupture and repair processes is helpful to understand how certain relational dynamics become destructive. According to Safran and Muran (2000), ruptures in the alliance can either be dramatic occurrences that inevitably alter the therapeutic climate, or subtle—sometimes even unnoticeable—tensions in the interaction. However, the clients in this study rarely considered minor tensions or small misattunements to be relational ruptures. On the contrary, clients welcomed a therapist who was able to challenge them and evoke difficult emotions, and small irregularities within the relationship (e.g., feeling overwhelmed or needing a break from intensive therapeutic work) were seen as a natural part of therapy. This is in line with the thinking of Gelso and Kline (2019), who argue that we tend to use the term ‘rupture’ too loosely, and enables us to distinguish between ruptures in the real relationship and the alliance. While ruptures in the alliance are inevitable, ruptures in the real relationship are less frequent but more damaging. These ruptures need explicit and immediate reparation (Gelso and Kline, 2019).

Hence, it seems that clients desire tension and tolerate small ruptures as long as the therapeutic relationship is strong (i.e., presence of togetherness, characterized by the client feeling safe, having faith in the therapist’s ability to help and feeling equal and worthy of help). Yet, larger relational ruptures have destructive potential if not addressed and resolved (Eubanks et al., 2019). The current study does not detail large relational ruptures, although some instances can be considered ruptures in the real relationship (e.g., in dyad 8, when the client feels that the therapist seriously underestimates her). Rather, the current study points to what happens when the real relationship fails to develop in the first place (dyads 4, 9 and 11, in particular). Instead of finding common ground, an empty space emerges between client and therapist. In this ‘no man’s land’, they keep one another at a distance, neither of them able to reach the other.

This space was exacerbated when the therapist pursued therapeutic effectiveness over the relationship. If the client did not feel safe, acknowledged or seen as a person, there was no room or contract for therapy. Accordingly, the relationship was restored when the therapist put aside technical aspects and focused exclusively on the real relationship, either through helping the client feel safe and relaxed, or redressing their sense of feeling unworthy, inferior or shameful. In this study, overwhelming fear and shame fueled relational distance. Our findings resonate with other qualitative research, confirming that feelings of fear and shame are common during initial sessions and need to be dealt with to successfully reach a real relationship and a collaborative working alliance (Lavik et al., 2018; Radcliffe et al., 2018; Kleiven et al., 2020). Further, serious ruptures or a lack of relationship became fatal when not addressed, as in dyads 4, 9, and 11. Here, both client and therapist suffer in solitude, as each is unaware of the other’s struggle.

Miller (2005) defines psychotherapy as an inherently moral practice, and places the client’s suffering at the core of therapeutic work. He further argues that most psychotherapy clients have a history of emotional harm inflicted by other people, sometimes by those whom they have trusted or loved. However, he continues, contemporary psychotherapy needs to acknowledge its moral dimension to rediscover a critical aspect of psychotherapeutic practice, namely that the therapeutic relationship functions as a psychological and moral restitution for the harm done to clients. Instead of taking in the client’s suffering and harvesting its potential for growth and change, current therapies risk ‘de-moralizing’ the suffering of the client through the use of general diagnoses, biomedical paradigms, and adherence to standardized treatment protocols not tailored to the individual client (Miller, 2004, 2005). In the current study, this moral dimension is foregrounded: encountering a new therapist raises several questions within the client (e.g., ‘do you really care for me?’, ‘do you understand me and where I come from?’, ‘are you there for me and can I trust you?’). This can be understood as the client questioning the therapist’s intentions and agenda, trying to figure out whether the therapist is morally capable of handling their story, vulnerability, and suffering. These internal processes are important for the formation of a real relationship and a truly collaborative working alliance. As Miller (2005) describes it, the moral dimension of clinical work emerges when the therapist pays attention to the client’s narrative, recognizes and responds to the moral concerns raised by the client, and helps restore faith in the healing capacities of the therapeutic relationship.

### Strengths and Limitations

A central asset of the current study is the dyadic perspective, integrating both sides of the story as well as providing a unique understanding of clients’ and therapists’ experiences of the same therapeutic endeavor. Another advantage is the combination of two sessions, enabling us to track the development of the relationship across time. Further strengths include the variation in therapist expertise, treatment methods, different clinics, and age and gender of participants.

Most importantly, the IPR method gives richness and depth to the data, bringing forth therapeutic moments that could easily have faded away and remained undiscovered in a traditional retrospective interview. Simultaneously, however, IPR is complex, demanding and exhausting for both interviewer and interviewee. Because it is time consuming, the IPR interviews did not allow for examination of the entire therapeutic session, meaning that vital moments may have been missed and left out. To compensate, we began each interview by asking the participant if there were any vital or striking moments that would be especially important to revisit during the interview. Quite often there were, and we were able to pay close attention to these moments.

As with other qualitative research, both rapport and a trusting researcher-participant relationship are vital in order to obtain rich, in-depth interviews. Having a second IPR interview was helpful in this regard, and we experienced increasing eagerness and engagement from participants in the second interview. To address response-bias, we strove for an open-minded, curious and accepting attitude. Further, interviews were conducted by a clinical psychologist (first author) and clinically trained psychology students. The last author closely supervised the initial interviews, and feedback was provided so that interview techniques could be enhanced.

Supplementing qualitative research with quantitative measures of the real relationship and/or the working alliance, or using outcome measures tracking the client’s response to therapy, could be beneficial for future studies on relational processes. Further, using the IPR method to investigate the very first therapy session could yield other interesting findings. In addition, it could be interesting to study other adjacent components of the therapeutic relationship using IPR, such as transference-countertransference (Gelso and Carter, 1994), which were beyond the scope of the present analysis.

Qualitative research does not aim to be generalizable to larger populations, but rather to the phenomenon in question (Levitt, 2021). The themes accounted for in this study were commonly described across all dyads. Micro-analytic research designs, such as IPR, are suitable for investigating relational and dyadic phenomena, and focusing on the dyad as a unit provides us with unique insights of clinical value. However, future studies with a wider range of therapies are needed to enhance our understanding of relational processes even further.

### Implications

The results from the current study illuminate how the therapeutic relationship serves as a necessary prerequisite for therapeutic work, and subsequently how these concepts mutually affect and depend on each other. However, we also found that relational processes can be disturbed and even demolished when the therapist focuses overly on technical aspects of therapy, rather than a collaborative and genuine relationship. Taken together with the massive research literature showing the relationship to be vital for successful psychotherapy, this has important implications for clinical psychology in a time where policy makers call for briefer, intensive and standardized evidence-based therapies. Regardless of therapeutic context, therapists need to be given the time and space necessary to form a strong relationship, and therapy needs to be tailored to the unique client to be effective.

## Conclusion

In this study, we explored the complex dyadic relational processes that either facilitated or hindered the formation of a therapeutic relationship in the first five sessions of psychotherapy. Our findings showed that a strong early relationship relied on a transition from initial fear and shame within the client, to a growing sense of safety, trust and togetherness with the therapist. As the therapeutic relationship unfolds, it appeared to start with an emerging ‘real relationship’, characterized by a genuine person-to-person relation, before it moved in the direction of a collaborative ‘working alliance’ in which both the client and therapist are actively engaged in the ongoing therapeutic work.

## Data Availability Statement

The datasets presented in this article are not readily available because this data material consist of in-depth qualitative interviews of both clients and therapists from the same therapeutic dyad. This warrant particular ethical considerations, as there is a risk that one participant can come to recognize the other from the transcripts. The transcripts are considered too sensitive and identifiable to be shared outside the research group. Requests to access the datasets should be directed to kristina.osland.lavik@helse-forde.no.

## Ethics Statement

The studies involving human participants were reviewed and approved by Regional etisk komité (REK VEST). The patients/participants provided their written informed consent to participate in this study (reference number 2015/2319).

## Author Contributions

KL was the project leader and main author of the article, and conducted 31 of 47 IPR interviews. AM and EK contributed to the analysis process. CM supervised and audited in all phases of the project. All authors contributed to the article and approved the submitted version.

## Funding

This project was supported and funded by Helse Førde Hospital Trust and Helse Vest Hospital Trust.

## Conflict of Interest

The authors declare that the research was conducted in the absence of any commercial or financial relationships that could be construed as a potential conflict of interest.

## Publisher’s Note

All claims expressed in this article are solely those of the authors and do not necessarily represent those of their affiliated organizations, or those of the publisher, the editors and the reviewers. Any product that may be evaluated in this article, or claim that may be made by its manufacturer, is not guaranteed or endorsed by the publisher.
